# Building optimism at the environmental science-policy-practice interface through the study of bright spots

**DOI:** 10.1038/s41467-018-05977-w

**Published:** 2018-08-28

**Authors:** Christopher Cvitanovic, Alistair J. Hobday

**Affiliations:** 1 CSIRO Oceans and Atmosphere, Hobart, TAS Australia; 2 Centre for Marine Socioecology, University of Tasmania, Hobart, TAS Australia

## Abstract

Effectively translating scientific knowledge into policy and practice is essential for helping humanity navigate contemporary environmental challenges. The likelihood of achieving this can be increased through the study of bright spots—instances where science has successfully influenced policy and practice—and the sense of optimism that this can inspire.

Successfully navigating contemporary environmental challenges, such as those encapsulated by the Sustainable Development Goals, requires the integration of new and evolving scientific knowledge into decision-making processes. Achieving demonstrable impacts on policy and practice, however, is not easy, and despite significant efforts from scientists and decision-makers alike, the uptake and integration of scientific knowledge into decision-making processes remains a significant challenge. Rather, evidence suggests that decision-makers primarily rely on experiential knowledge in isolation from evidence-based science, limiting the potential success of policy and management decisions with downstream consequences for societal well-being and prosperity.

Accordingly, the past two decades have seen calls for scientists to find new ways of engaging more effectively with decision-makers^1^, as well as demonstrate the tangible and real world impacts arising from their research. As a result, there has been an increase in efforts to identify new pathways to support evidence-informed decision-making^2^. For the most part, such efforts have focused on documenting and understanding situations where science has failed to inform policy or practice, and in turn, identify the barriers that prevent the successful integration of these domains. While the study of failures and challenges is an important first step in the identification of a problem, this approach has also inevitably contributed towards the widespread adoption and institutionalisation of the mantra ‘science-policy gap’, which now dominates much of the public and academic discourse in this space^3^.

We contend that the continued propagation of this mantra is counterproductive to improving the relationship between environmental science, policy and practice. For example, the dominance of negative terminology such as ‘gap’ can increase anxiety in scientists (and particularly early career scientists^4^) seeking to influence policy or practice, disempowering them from even trying. Similarly, the use of the term ‘gap’ validates the misleading and outdated notion that scientists and decision-makers are distinct groups of individuals divided by a range of unsurmountable cultural and epistemological differences, rather than recognising their interdependency and shared values in the pursuit of a common goal – environmental sustainability and conservation.

We argue that a shift in the academic study of science–policy–practice interfaces is needed towards the study of bright spots— outliers that perform significantly better than would be expected^5^. In this case, bright spots would represent situations whereby environmental science has successfully influenced policy and/or practice, despite the many documented challenges and barriers. We believe that the systematic documentation of success will help to establish a new mantra of optimism, in turn conferring a range of benefits to both individuals and teams and increasing the likelihood that science will influence and impact on policy and practice. We also suggest that the study of bright spots is essential for identifying new and improved strategies of reconciling the use of environmental science in policy and practice. Here we draw on other fields (e.g. psychology and organisational behaviour), sectors (e.g. business) and our personal experiences to explore the value of optimism in this context, and identify the key lessons and principles that can be used to increase the likelihood that environmental science will positively influence and impact policy and practice.

## The power of optimism

The benefits associated with optimism—which we define as the generalised positive expectancy that one will experience good outcomes^6^—have been explored extensively across different disciplines and sectors. For example, the field of psychology offers many insights relating to the power of optimism as it relates to the relationship between science, policy and practice. Individuals with an optimistic outlook routinely maintain higher levels of psychological well-being during times of stress than those who are less optimistic^7^. As a result, these individuals are more likely to accept the reality of stressful and challenging situations, and take direct action to overcome the adversity and attain their goals^8^. Therefore on an individual level a sense of optimism can inspire action, help individuals to navigate and cope with challenging situations and increase their likelihood of achieving impacts on policy and practice.

In a similar way optimism has been shown to underpin effective team coordination and collaboration^9^, by encouraging the full engagement of teammates with regard to interactions, knowledge sharing and cooperation^10^. As a result, optimism among teams also works to minimise potential sources of conflict that may arise. In contrast, pessimism has been shown to increase anxiety among team members, leading to increased competition and poor team performance. In light of the growing evidence in the environmental sector for the need for closer collaboration among scientists (across disciplinary boundaries) and decision-makers, the establishment of a more optimistic outlook, therefore, may be a critical factor that can increase the likelihood of success.

Optimism is also closely related to creativity at both the individual and team levels^11^. Creativity is important as it is closely linked with innovation and problem solving, and thus is a key influence on the ability of individuals and teams to achieve their goals in the face of complexity and uncertainty^12^. Innovation may be particularly important for improving the relationship between environmental science, policy and practice, given that many of the barriers identified at this interface are deeply entrenched within our existing academic and decision-making institutions and cultures^2^, and the identification of innovative approaches to improve the use of science in policy and practice are needed. Further, innovation is considered critical for the development of transformative solutions to modern day environmental challenges. Therefore, establishing a more optimistic outlook at the interface of environmental science, policy and practice is an important next step for the development of sustainability solutions and enabling evidence-informed decision-making.

## Bright spots at the interface of science, policy and practice

One way to achieve an optimistic outlook is via the systematic documentation of bright spots, instances where science has successfully influenced policy and/or practice. However, the study of bright spots can not only help inspire optimism—their study can also help elucidate new ways of successfully linking environmental science to policy and practice. By their very nature, outliers such as bright spots deviate from expectations and consequently can provide novel insights for responding to complex challenges^5^. While, based on our experiences these science–policy–practice bright spots are common and wide-spread, they are seldom documented.

One example of such success involved the development of intervention options to safeguard an iconic bird species, the Shy Albatross, which was showing signs of population declines under global warming and from disease (Table 1). It was clear that existing approaches to management focussed on threat abatement were not sufficient, and alternate responses were needed— namely, interventions. Scientists worked closely with managers and identified a range of potential options that were assessed according to their cost–benefit-risk. The first of these interventions, the treatment of albatross chicks for ecto-parasites, was subsequently field tested^13^ and found to improve chick survival rates by 10%. In light of this success, this intervention has been expanded and new interventions are now also being field tested (e.g. use of artificial nests to offset climate impacts).

**Table 1:** Detailed overview of two science–policy–practice bright spots, including the science need, research outcomes and impacts achieved

Another example involved generating new scientific knowledge to empower a marine industry—the Southern Bluefin Tuna (SBT) fishery in Australia’s Great Australian Bight—to overcome challenges posed by climate change (Table 1). Specifically, a dramatic change in the distribution of SBT compromised the ability of the fishery to efficiently locate and harvest the species, with subsequent impacts on commercial viability, as well as the upstream and downstream supply chain links. In partnership with industry representatives, scientists developed a new seasonal forecasting system to project the likely distribution of SBT several months into the future^14^. These forecasts are now delivered daily via an industry-specific website tailored to user needs, and have been shown to assist fishers to efficiently catch SBT under variable climatic conditions (Table 1).

## Lessons from the study of bright spots

**Fig. 1:**
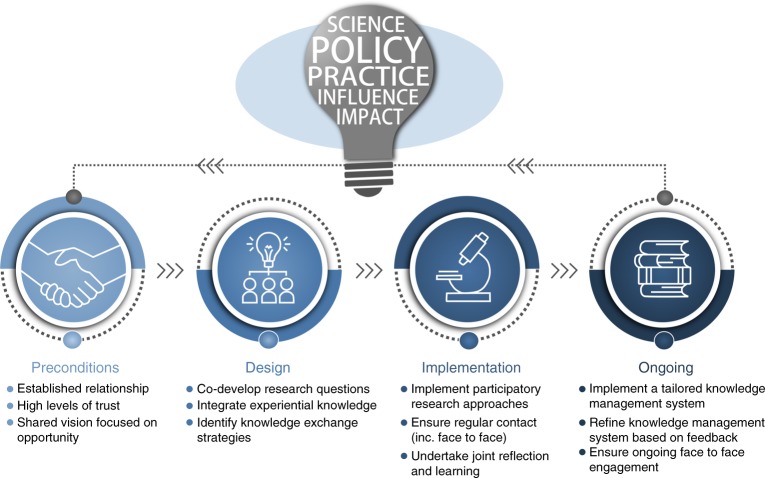
Key principles underpinning success across science–policy–practice bright spots. These guidelines are based on the experiences of the two bright spots described in Table 1 (Shy Albatross and Southern Bluefin Tuna)

Looking at the lessons learnt across these (and other) case studies allows for the identification of key principles that underpin success—which in turn can be used to help guide the efforts of other scientists seeking to influence policy and/or practice^15^. For example, in each case study, a precondition to the research activity was the development of strong and trusted relationships with key stakeholders (Fig. 1). These relationships then formed the basis for the joint design of the research activity, which involved the co-development of research questions that accounted for the experiential knowledge of managers, as well as the identification of specific knowledge-exchange strategies to be implemented throughout the research process (Fig. 1). The research activity was then implemented using participatory research approaches (e.g. co-production), whereby decision-makers were active participants in each phase of the scientific research process, and supplemented by mechanisms that ensured regular contact among scientists and end-users that facilitated joint reflection and learning throughout the process. Following the completion of research activity, mechanisms were set in place to ensure ongoing communication and engagement among scientists and the relevant end-users, for example, via the establishment and refinement of a tailored knowledge management system (Fig. 1).

While these principles are based on our subjective experiences in these processes, they highlight how the study of bright spots can help to elucidate the key principles for improving the impact of environmental science on policy and practice. Further, based both on our personal experiences as well as previous studies seeking to identify the key principles underpinning the impact of environmental science on policy and practice^15^, we believe that these principles will be largely applicable irrespective of discipline, geography and/or scale. For example, the empirical analysis of interviews with 32 researchers and stakeholders across 13 different environmental management research projects across the United Kingdom identified five principles for the effective practice of knowledge exchange among scientists and decision-makers^16^. The key principles identified through this study reflect those described in Fig. 1, such as the need for strong trusted relationships among actors, the cogeneration of knowledge through participatory research approaches and the need for joint learning and reflection.

To conclude, increasing the influence and impact of environmental science on policy and practice necessitates moving beyond the ongoing diagnosis of challenges and barriers—towards the study of bright spots. As highlighted through our examples, the systematic study of bright spots will help to identify the key principles underpinning success, and allow for the development of more effective strategies for successfully navigating the interface of science, policy and practice. Even more importantly, we contend that the systematic study of bright spots will be essential for moving beyond the existing mantra surrounding the interface of science, policy and practice towards a new mantra of optimism—one that inspires hope and empowers scientists and decision-makers to continue to strive for new ways of working together for a sustainable future.
